# Reduced quadriceps deficit following dry arthroscopy compared to standard fluid arthroscopy at the time of MPFL reconstruction

**DOI:** 10.1002/jeo2.70367

**Published:** 2025-07-13

**Authors:** Clément Favroul, Robert A. Magnussen, Cécile Batailler, Sébastien Lustig, Elvire Servien

**Affiliations:** ^1^ Orthopaedics Surgery and Sports Medicine Department, FIFA Medical Center of Excellence, Croix‐Rousse Hospital, Hospices Civils de Lyon Lyon North University Hospital Lyon France; ^2^ OSU Sports Medicine Sports Health and Performance Institute The Ohio State University Columbus Ohio USA; ^3^ Univ Lyon, Claude Bernard Lyon 1 University Lyon France; ^4^ LIBM‐EA 7424, Interuniversity Laboratory of Biology of Mobility Claude Bernard Lyon 1 University Lyon France

**Keywords:** arthrogenic muscle inhibition, dry arthroscopy, knee, MPFL, quadriceps deficit, RPD

## Abstract

**Purpose:**

Recurrent patellar dislocations (RPDs) are prevalent, particularly among adolescents. The ‘Menu à la carte’ technique facilitates personalized management of each patient through reconstruction of the medial patellofemoral ligament (MPFL) while simultaneously addressing other anatomical factors that may contribute to instability. Arthroscopy, frequently performed prior to stabilization surgery to identify and treat intra‐articular pathology, carries specific risks. Fluid arthroscopy can increase post‐operative effusion, while dry arthroscopy may expose cartilage to potential damage due to the absence of fluid, increasing friction and thermal effects. Concurrent knee arthroscopy to evaluate and address intra‐articular pathology at the time of MPFL may contribute to post‐operative quadriceps deficits that can significantly impact recovery. Therefore, this study aimed to evaluate and compare quadriceps recovery following either traditional fluid or dry arthroscopy at MPFL reconstruction. The hypothesis was that MPFL reconstruction with fluid arthroscopy would result in more significant quadriceps deficiency, more effusions, and decreased early post‐operative knee flexion compared to the dry arthroscopy group.

**Methods:**

This retrospective study analyzed 66 patients who underwent MPFL reconstruction for RPD between February 2020 and February 2024. Exclusion criteria included trochleoplasty, tibial tubercle osteotomy, or missed isokinetic tests beyond 6 months post‐operatively. Patients underwent fluid arthroscopy until September 2021 and dry arthroscopy thereafter. Post‐operative follow‐up at 6 weeks recorded range of motion, patellar apprehension and clinical diagnosis of knee effusion, while isokinetic testing at 4–6 months compared peak torque and muscle strength between the fluid and air arthroscopy groups.

**Results:**

Isokinetic tests revealed a significant deficit in quadriceps strength of the operated knees during MPFL reconstructions performed under fluid compared to those performed dry, both at concentric 60°/s (73.3 ± 33.8 vs. 95.5 ± 39.5 N m/kg; *p* = 0.02), and at concentric 240°/s (53.6 ± 27.0 vs. 66.5 ± 28.7 N m/kg; *p* = 0.04) between 4 and 6 months post‐operative. The limb symmetry index (LSI) showed a significant quadriceps strength deficit in the fluid arthroscopy group compared to the dry arthroscopy group, both at concentric 60°/s (63.8 ± 18.0 vs. 75.2 ± 17.2; *p* = 0.01) and 240°/s (69.6 ± 19.8 vs. 78.5 ± 13.6; *p* = 0.04). The fluid arthroscopy also demonstrated an increased incidence of post‐operative effusion (47% vs. 19%, *p* = 0.01) and decreased knee flexion (129.7 ± 13.9° vs 119.3 ± 16.3°, *p* = 0.01) when compared to the dry arthroscopy group at 6 weeks post‐operative.

**Conclusion:**

This study has demonstrated a significant quadriceps strength deficit in isokinetic tests following fluid arthroscopy compared to dry arthroscopy as well as a higher incidence of effusion and flexion deficit post‐operative. These findings highlight the potential for dry arthroscopy to assess intra‐articular pathology during MPFL reconstruction.

**Level of Evidence:**

Level III, retrospective, case–control study.

**Abbreviations**
ACL: anterior cruciate ligament
AMI: arthrogenic muscle inhibition
BMI: body mass index
CDI: Caton–Deschamps index
H/Q: hamstrings/quadriceps
LSI: limb symmetry index
MPFL: medial patellofemoral ligament
ROM: range of motion
RPD: recurrent patellar dislocation

## INTRODUCTION

Patellar instability is common in adolescents, with incidences of 5.8–29 per 100,000 in the 10–17 age group [36]. Lateral dislocations, often occurring during non‐contact play [7], are linked to anatomic risk factors such as ligamentous laxity, tibial torsion, femoral anteversion, patella alta and trochlear dysplasia [5, 12, 36]. Medial patellofemoral ligament (MPFL) injuries frequently follow dislocations, increasing the risk of instability as the MPFL is the primary soft tissue stabilizer [31].

Managing patellar instability can be challenging, given the multiple anatomical contributors to instability and available treatment options [5]. MPFL reconstruction has become the mainstay of surgical treatment of recurrent patellar dislocation (RPD), with bony procedures also considered according to the ‘Menu à la carte’ technique [5], which facilitates personalized management of each patient through reconstruction of the MPFL while simultaneously addressing other anatomical factors that may contribute to instability

Chondral damage is common in RPD, resulting from trauma, instability‐related loading and anatomical abnormalities [11, 18, 23]. Acute injuries, including osteochondral fractures and fissures, occur in 70–80% of cases [11, 18], with loose bodies in 31–58% of first dislocations [9, 30, 34]. Given the relatively high prevalence of intra‐articular injuries, diagnostic arthroscopy is often performed to identify and treat them at the time of patellar stabilization surgery.

MPFL reconstruction recovery can be challenging, often due to post‐operative quadriceps deficits [4]. Its origins are multifaceted, encompassing neural, arthrogenic, and muscular factors. Neural factors implicate alterations in brain cortical and corticospinal activity following knee surgery, leading to disrupted quadriceps control [24]. Arthrogenic influences, such as pain and effusion, contribute to dysfunction through a reflexive response aimed at protecting the joint, known as arthrogenic muscle inhibition (AMI) [6, 32, 33]. This phenomenon limits muscle activation, exacerbating quadriceps weakness. Additionally, muscular factors play a significant role, with rapid‐onset muscle atrophy being a common consequence of disuse, immobilization and non‐weight‐bearing post‐surgery [35]. The interaction of these factors highlights the need for comprehensive rehabilitation strategies targeting all three components to optimize recovery.

Dry arthroscopy is a less commonly used technique compared to traditional fluid arthroscopy. While fluid arthroscopy involves distending the joint with a saline solution to create space for visualization and instrumentation, dry arthroscopy is performed without fluid. Despite its potential advantages, such as reduced fluid extravasation, improved visualization and lower cost, dry arthroscopy is relatively understudied and less frequently performed [1, 10, 16, 25, 26, 28]. There is limited research comparing the outcomes of dry arthroscopy with those of traditional fluid arthroscopy, particularly in the context of capsular distension, which could contribute to post‐operative quadriceps weakness [32].

The aim of this study is to compare quadriceps strength, knee range of motion (ROM), and the presence of a knee effusion following isolated MPFL reconstruction in conjunction with either fluid or dry arthroscopy. The hypothesis was that MPFL reconstruction with fluid arthroscopy would result in more significant quadriceps deficiency, more effusions, and decreased early post‐operative knee flexion compared to the dry arthroscopy group.

## MATERIALS AND METHODS

### Ethical approval

All procedures were performed in accordance with the ethical standards of the institutional and/or national research committee, the 1964 Helsinki Declaration and its later amendments, or comparable ethical standards. Data collection and analysis were carried out in accordance with MR004 Reference Methodology from the Commission Nationale de l'Informatique et des Libertés (Ref. 2229975V0) obtained on 6 May 2023. The study was registered and filed on the Health Data Hub website.

### Patients

**Figure 1:**
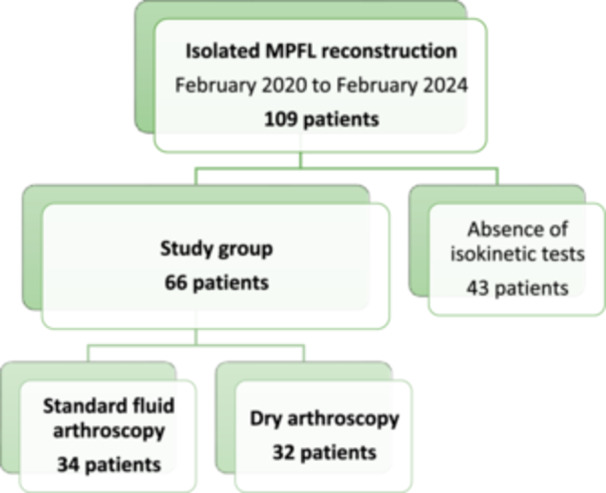
Study's flow chart.

This retrospective, single‐centre study included 66 patients who underwent isolated MPFL reconstruction for confirmed RPD. Patients who underwent MPFL reconstruction with trochleoplasty, tibial tubercule osteotomy, or had a history of knee surgery were not included in the study. All surgeries were conducted by the same senior surgeon between February 2020 and February 2024. Of the 109 isolated MPFL reconstruction surgeries performed during this period, 43 patients were excluded from the study as they did not undergo isokinetic testing within 6 months following MPFL reconstruction (Figure 1).

### Surgical technique

All procedures began with arthroscopy to evaluate trochlear dysplasia and to identify and treat any chondral lesions. Between February 2020 and 1 September 2021, all arthroscopy was conducted using standard fluid arthroscopy. After 1 September 2021, arthroscopy was performed with air. In all cases, a standard anterolateral portal was created, followed by the insertion of an obturator and then the arthroscope into the knee joint. Next, for dry arthroscopy, two 20 cc syringes of air are injected into the knee to achieve capsular distension. If necessary, an additional 20cc syringe of air was added. During the standard fluid arthroscopy, distension was achieved using saline solution under a pressure of 50 mmHg delivered by a pump. The arthroscopy was then performed to identify and classify chondral lesions using the International Cartilage Repair Society classification, assess patellar tracking and treat any intra‐articular pathology.

MPFL reconstruction was performed the same technique for both groups and was performed under a thigh tourniquet, with only the diagnostic arthroscopy differing. The reconstruction of the MPFL was performed using the gracilis autograft according to the technique described [19].

### Rehabilitation

Post‐operatively, patients were allowed full weight‐bearing immediately with the use of a hinged brace for the first few days until effective quadriceps activation and unrestricted passive ROM were achieved. Rehabilitation included concentric quadriceps and hamstring strengthening exercises in closed kinetic chains for the first 45 days, with no restriction on flexion. Weighted squats and dynamic quadriceps strengthening exercises were permitted after 3 months, along with jogging. Recently developed rehabilitation protocols specifically to address AMI were not implemented during the study period to avoid confounding the effect of arthroscopy techniques. There were no activity restrictions beyond 3 months, and patients were permitted to return to sports once pain and swelling had resolved and they felt ready to resume activity.

### Clinical and radiographic data

All patients underwent standardized preoperative physical examination, during which ROM, patellar apprehension and patellar tracking (present or absent J‐sign) were recorded. Radiographs were evaluated using Centricity Universal Viewer Zero Footprint (version 6.0 SP7.0.2—GE Healthcare). Trochlear dysplasia was classified using Dejour's classification [5], and patellar height was assessed by measuring the Caton–Deschamps index (CDI) [3]. Post‐operative follow‐up at 6 weeks after surgery was also standardized, during which ROM was measured with a goniometer, patellar apprehension was assessed, and the presence of knee effusion was evaluated using the patellar tap test, all recorded by the same senior surgeon.

### Isokinetic tests

Isokinetic testing was conducted by an independent examiner (a sports medicine physician) between 4 and 6 months following the operation. The Con‐Trex® (Physiomed Elektromedizin AG) isokinetic dynamometer was employed for isokinetic assessment. Patients followed a standardized protocol that commenced with a 15‐min warm‐up on a stationary bike, starting with the non‐operative side for each testing phase. Prior to each test, participants completed submaximal trials to familiarize themselves with the procedures and for training purposes.

The assessment began with the concentric phase, involving three repetitions at 60°/s followed by three at 240°/s for each knee. The ROM spanned 70° (20–90°). Subsequently, eccentric tests were conducted with three maximal repetitions at 30°/s. Peak torque measurements, recorded in Newton‐metres (Nm), were normalized to body weight (Nm/kg) and recorded for each muscle group. Additionally, the hamstrings/quadriceps (H/Q) ratio and limb symmetry index (LSI) between the healthy knee and the operated knee were computed for each speed. The primary outcome was quadriceps strength, while the secondary outcome focused on hamstring strength. Normalized strength for each muscle group, H/Q ratio and LSI were compared between the fluid and air arthroscopy groups.

### Statistical analysis

The statistical analysis was performed using XLstat (version 2015.1, Addinsoft). Continuous variables were averaged and reported with standard deviation, minimum and maximum. Categorical variables were represented by their sum and percentage. Significant differences were calculated using the Student's *t* test for continuous variables and the chi‐square test for categorical variables. A *p* value < 0.05 was considered statistically significant. A power analysis was conducted using XLstat (version 2015.1, Addinsoft) to determine the sample size required to detect a 20% difference in quadriceps strength between the fluid arthroscopy control group and the dry arthroscopy group. These analyses found that 62 total participants (31 per group) would be necessary to achieve 80% power to detect the above difference with an alpha of 5.0% (*p* < 0.05) using a two‐tailed test.

## RESULTS

Pre‐operatively, the two groups were similar in terms of age, gender, body mass index (BMI), ROM, patellar tracking (j‐sign), CDI, Dejour classification and chondral lesions identified during arthroscopic exploration (Table 1).

**Table 1:** Patient and demographic data for fluid and dry arthroscopy groups.

Parameters *N* (%) mean ± SD [min; max]	Fluid arthroscopy group, *N* = 34	Dry arthroscopy group, *N* = 32	Significance
Gender	F: 27 (79%)	F: 21 (66%)	0.32
	M: 7 (21%)	M: 11 (34%)	
Age (y‐o)	25.4 ± 7.7 [13; 40]	24.3 ± 8.4 [12; 49]	0.59
BMI (kg/m^2^)	23.1 ± 4.7 [14.5; 38.6]	24.1 ± 5.1 [17.6; 39]	0.44
Preoperative ROM			
Extension	3.8 ± 3.9 [−5; 10]	5.6 ± 6.1 [−5; 20]	0.16
Flexion	138.8 ± 8.5 [120; 150]	134.3 ± 16.7 [90; 160]	0.13
Preoperative J‐sign	Yes: 8 (24%)	Yes: 9 (28%)	0.67
	No: 26 (76%)	No: 23 (72%)	
Caton–Deschamps index	1.18 ± 0.14 [0.9; 1.38]	1.16 ± 0.14 [0.7; 1.4]	0.43
Dejour classification of trochlear dysplasia			
A	11 (33%)	9 (28%)	
B	16 (47%)	15 (47%)	0.76
C	6 (17%)	6 (19%)	
D	1 (3%)	2 (6%)	
Chondral lesions according to ICRS			
Trochlea	ICRS 1: 1; ICRS 2: 3	ICRS 1: 0; ICRS 2: 2	0.20
	ICRS 3: 1; ICRS 4: 0	ICRS 3: 3; ICRS 4: 1	
Patella	ICRS 1: 0; ICRS 2: 10	ICRS 1: 1; ICRS 2: 6	0.47
	ICRS 3: 0; ICRS 4: 0	ICRS 3: 5; ICRS 4: 0	

Abbreviations: BMI, body mass index; F, female; ICRS, International Cartilage Repair Society; M, male; Max, maximum; Min, minimum; NS, non‐significant; ROM, range of motion; SD, standard deviation.

The clinical examination of patients conducted at 6 weeks post‐operatively revealed a significantly higher incidence of clinical effusions in the fluid arthroscopy group compared to the dry arthroscopy group (47% vs. 19%, *p* = 0.01) (Table 2). There were no significant differences observed between the groups regarding knee extension, but flexion was significantly greater in the dry arthroscopy group than in the fluid arthroscopy group (129.7 ± 13.9° vs. 119.3 ± 16.3°, *p* = 0.01). No significant difference was found between the groups regarding continued patellar apprehension (1 positive [3%] in the fluid group vs. 2 [6%] in the dry group) (Table 3).

**Table 2:** Patient and demographic data for fluid and dry arthroscopy groups at 6 weeks post‐operatively.

Parameters *N* (%) mean ± SD [min; max]	Fluid group, *N* = 34	Dry group, *N* = 32	*p* **value**
Days since surgery	50.4 ± 6.3	45.2 ± 5.8	0.31
Knee effusion	Yes: 16 (47%)	Yes: 6 (19%)	0.01
	No: 18 (53%)	No: 26 (81%)	
Post‐operative ROM			
Extension	0.3 ± 1.7 [−5; 5]	1.2 ± 3.3 [−3; 10]	0.17
Flexion	119.3 ± 16.3 [90; 140]	129.7 ± 13.9 [90; 150]	0.01
Apprehension test	Positive: 1 (3%)	Positive: 2 (6%)	0.53
	Negative: 33 (97%)	Negative: 30 (94%)	

Abbreviations: Max, maximum; Min, minimum; NS, non‐significant; ROM, range of motion; SD, standard deviation.

**Table 3:** Isokinetic strength test results and H/Q ratio.

Parameters mean ± SD [min; max]	Fluid group healthy side	Dry group healthy side	*p* **value**	Fluid group operated side	Dry group operated side	*p* **value**
Quadriceps (N m/kg)						
Concentric 60°/s	106.4 ± 31.1 [62; 195]	124.9 ± 45.4 [58; 250]	0.07	73.3 ± 33.8 [29; 154]	95.5 ± 39.5 [43; 210]	0.02
Concentric 240°/s	75.7 ± 30.2 [38; 190]	85.7 ± 33.7 [37; 161]	0.21	53.6 ± 27.0 [27; 167]	66.5 ± 28.7 [24; 140]	0.04
Hamstrings (N m/kg)						
Concentric 60°/s	67.2 ± 25.4 [36; 132]	72.9 ± 22.7 [39; 124]	0.34	60.2 ± 22.8 [36; 122]	68.0 ± 21.2 [30; 118]	0.16
Concentric 240°/s	50.3 ± 20.9 [17; 99]	51.8 ± 17.1 [26; 95]	0.74	48.4 ± 20.3 [19; 99]	50.4 ± 18.8 [16; 97]	0.68
Eccentric 30°/s	93.0 ± 25.1 [52; 152]	86.4 ± 29.1 [43; 153]	0.35	76.1 ± 24.7 [46; 144]	79.7 ± 24.5 [28; 144]	0.58
H/Q ratio (%)						
Concentric 60°/s	66.0 ± 24.6 [34; 143]	59.7 ± 9.6 [48; 82]	0.17	90.5 ± 37.0 [44; 200]	72.4 ± 22.2 [13; 128]	0.02
Concentric 240°/s	69.6 ± 27.2 [23; 134]	62.4 ± 17.0 [35; 116]	0.20	92.4 ± 36.4 [27; 187]	74.7 ± 17.6 [48; 116]	0.01

Abbreviations: H, Hamstrings; Max, maximum; Min, minimum; NS, non‐significant; Q, Quadriceps; SD, standard deviation.

The fluid arthroscopy group had a mean delay of 5.5 ± 0.4 months (range: 4.3–6.0) between surgery and isokinetic testing, while the dry arthroscopy group had a mean delay of 5.4 ± 0.5 months (range: 4.2–5.9) (*p* = 0.76). The analysis of isokinetic tests (Table 3) revealed a significant deficit in quadriceps strength of the operated knees during MPFL reconstructions performed under fluid compared to those performed dry, both at concentric 60°/s (73.3 ± 33.8 vs. 95.5 ± 39.5 N m/kg; *p* = 0.02), and at concentric 240°/s (53.6 ± 27.0 vs. 66.5 ± 28.7 N m/kg; *p* = 0.04). There was no significant difference in hamstring strength between patients who underwent arthroscopy with fluid versus dry, whether tested concentrically at 60°/s or 240°/s, or eccentrically at 30°/s.

The H/Q ratios (Table 3) of the operated knees with significantly higher the fluid arthroscopy group both at concentric 60°/s (90.5 ± 37.0 vs. 72.4 ± 22.2; *p* = 0.02), and at concentric 240°/s (92.4 ± 36.4 vs. 74.7 ± 17.6; *p* = 0.01). There was no difference in H/Q ratios of healthy knees at concentric speeds of 60°/s and 240°/s.

Regarding the LSI comparing the operated knee to the healthy knee, there was a significant deficit in quadriceps strength in the fluid arthroscopy group compared to the dry arthroscopy group, both at concentric 60°/s (63.8 ± 18.0 vs. 75.2 ± 17.2; *p* = 0.01), and at concentric 240°/s (69.6 ± 19.8 vs. 78.5 ± 13.6; *p* = 0.04) (Table 4). There was no significant deficit in hamstring LSI between the fluid and dry arthroscopy groups at concentric speeds of 60°/s and 240°/s.

**Table 4:** Isokinetic strength test results for limb symmetry index (LSI) between healthy knee versus operated knee in fluid group versus dry group.

LSI mean ± SD [min; max]	Fluid group operated/healthy	Dry group operated/healthy	*p* **value**
Quadriceps deficit (%)			
Concentric 60°/s	63.8 ± 18.0 [34; 95]	75.2 ± 17.2 [29; 100]	0.01
Concentric 240°/s	69.6 ± 19.8 [13; 99]	78.5 ± 13.6 [38; 97]	0.04
Hamstrings deficit (%)			
Concentric 60°/s	84.0 ± 13.1 [36; 100]	83.5 ± 14.2 [40; 99]	0.87
Concentric 240°/s	83.8 ± 13.8 [41; 100]	82.2 ± 14.1 [45; 100]	0.65

Abbreviations: Max, maximum; Min, minimum; NS, non‐significant; SD, standard deviation.

## DISCUSSION

The most important finding of this study is that fluid arthroscopy is associated with a significant quadriceps strength deficit in isokinetic tests compared to dry arthroscopy, particularly at concentric 60°/s and 240°/s. Additionally, patients in the fluid arthroscopy group exhibited a higher incidence of post‐operative effusion and a greater limitation in knee flexion at 6 weeks post‐operatively. These findings suggest that dry arthroscopy may offer advantages in MPFL reconstruction by reducing post‐operative muscle weakness, minimizing joint effusion and preserving early knee mobility.

Persistent quadriceps weakness is important following MPFL reconstruction. The outcomes of this surgery are generally good, with more than 90% of patients returning to sports according to a meta‐analysis by Platt et al. [27]. However, the return to sports can be prolonged, averaging nearly 7–8 months post‐surgery for isolated MPFL reconstruction [17, 20, 27]. One of the accepted algorithms for returning to sports after MPFL reconstruction is the one developed by Ménétrey et al. [22]. With its comprehensive criteria (including no pain, no effusion, no patellofemoral instability, full ROM, nearly symmetrical strength (85–90%), and excellent dynamic stability). This algorithm emphasizes the importance of addressing quadriceps deficits, which must be reduced to sub‐pathological threshold values to ensure a safe return to activity without risking the knee.

Numerous studies highlight persistent quadriceps deficits in isokinetic tests following MPFL reconstruction. For instance, Hysing‐Dahl et al. [15] reported a 28% quadriceps asymmetry, with only 14% of patients meeting all RTS clearance criteria at this time point. These results align with our findings, demonstrating a quadriceps strength imbalance deficit, of over 36% at concentric 60°/s and over 30% at concentric 240°/s in the fluid arthroscopy group.

Few studies have examined outcomes of dry arthroscopy of the knee. Studies conducted on dry arthroscopy have mainly focused on the use of CO_2_ to achieve sufficient capsular distension [1, 26, 28]. Regarding intraoperative visualization, studies have shown that dry or gas‐assisted arthroscopy provides comparable, visibility of intra‐articular structures compared to standard fluid arthroscopy [1, 16]. The absence of fluid eliminates the distortion and reflection artefacts often caused by liquid media, allowing for a clearer view of cartilage integrity and subtle chondral lesions. Nonetheless, visualization can be more challenging in the presence of intra‐articular bleeding, where the absence of lavage may reduce clarity. Some concerns persist regarding the potential complications of dry arthroscopy, particularly related to cartilage health. The lack of fluid medium may increase friction and heat production during the use of shavers or radiofrequency devices, potentially exposing cartilage to mechanical or thermal injury. These risks require careful surgical technique and proper instrument handling to mitigate complications.

Other studies evaluating dry arthroscopy have concentrated on cartilage grafts, especially on autologous chondrocyte implantation systems [25]. Indeed, the use of a matrix requires a dry environment, for which CO_2_ is suitable. However, none of these studies have conducted clinical tests to evaluate functional outcomes in these patients. To our knowledge, this is the first study to demonstrate that diagnostic air arthroscopy results in reduced post‐operative quadriceps deficits during isokinetic tests compared to traditional fluid arthroscopy.

Several authors have previously highlighted quadriceps deficits following arthroscopic surgeries [2], including meniscectomies [13], autologous chondrocyte implantation [8], anterior cruciate ligament (ACL) reconstructions [14, 32] and even in MPFL reconstruction [21]. This study identifies the role of capsular distension by fluid during arthroscopy and its association with isolated quadriceps strength deficits post‐surgery. Indeed, capsular distension with air versus fluid was the only difference between the two groups in this study. Both the reconstruction technique and the rehabilitation protocols were held constant throughout, ensuring consistency. Notably, no significant differences were observed between the two healthy knees groups.

In terms of clinical outcomes, the study revealed a significant and clinically relevant difference in knee flexion and effusions at the 6‐week post‐operative mark, with the air arthroscopy group demonstrating 10° more flexion and reduced effusions compared to the fluid arthroscopy group. While there is currently limited research on effusions following MPFL reconstructions, the correlation between effusion size and knee flexion is consistent with existing literature, which suggests that draining hemarthrosis can lead to increased flexion [29].

Joint effusions and distension can also trigger a protective mechanism in surrounding muscles, thus causing AMI. Consequently, the quadriceps muscle may exhibit weakness and reduced activation, complicating the rehabilitation process. Understanding the potential for AMI development is crucial for devising effective rehabilitation strategies and optimizing patient outcomes following surgery. Sonnery‐Cottet et al. [32, 33] investigated AMI following ACL reconstructions and found that draining hemarthrosis reduced quadriceps inhibition. This correlation is consistent with the findings of this study, which state that a dry arthroscopy leads to less early effusion and subsequent quadriceps inhibition.

Further investigation into the underlying mechanisms driving the development of knee effusions, limited flexion, and persistent quad weakness following dry versus traditional arthroscopy would likely provide valuable insights for optimizing patient outcomes following knee arthroscopy. Understanding and addressing these issues are crucial steps towards restoring quadriceps function and enhancing patient outcomes following knee surgery. This knowledge could be invaluable for tailoring the rehabilitation protocols of patients undergoing MPFL reconstruction among other procedures.

In this study, we observed a trend towards greater quadriceps strength at 60°/s in the dry group for the healthy knee. While this finding raises a theoretical concern regarding the potential influence of gender on the results, it is important to highlight that this tendency was not present in any of the other comparisons, including at 240°/s and for hamstring strength. If the observed difference were truly due to the higher proportion of males in the dry group, we would expect to see a similar trend in hamstring strength, which was not the case. Furthermore, there was no significant difference in the gender distribution (34% vs. 21%) or age between the groups, both of which suggest that the observed trend is more likely due to the natural variability inherent in muscle strength testing rather than demographic imbalances.

There are limitations to this study. It is important to note that this study was performed at a single‐centre study utilizing a single surgical technique, which may not be generalizable to all situations. Further, the retrospective nature of the analysis may introduce biases and confounding variables that may not be adequately controlled. The relatively small sample size also precludes any subgroup analysis. However, meeting the required sample size based on the power analysis confirmed the achievement of the necessary power for the outcomes of interest. Moreover, more than one third of potentially eligible patients were not included in the study because isokinetic tests were not conducted or were conducted outside the designated timeframe post‐operatively. However, the isokinetic tests were performed according to standardized protocols, using machines whose reliability and accuracy have been validated in the past, thus ensuring reliable results.

Based on this study, dry arthroscopy is a safe alternative to traditional fluid arthroscopy during MPFL reconstruction when intra‐articular exploration is needed. It is best suited for scheduled procedures without active bleeding risks and may be less effective in cases with hemarthrosis or significant synovitis. While it provides comparable visualization, meticulous haemostasis is crucial, and motorized instruments should be used cautiously to avoid cartilage damage. Adapting to the air medium requires experience, and initial use should be limited to skilled arthroscopists. Dry arthroscopy is particularly useful for preserving quadriceps function.

## CONCLUSION

This study has demonstrated a significant quadriceps strength deficit in isokinetic tests following fluid arthroscopy compared to dry arthroscopy as well as a higher incidence of effusion and flexion deficit post‐operative. These findings highlight the potential for dry arthroscopy to assess intra‐articular pathology during MPFL reconstruction.

## AUTHOR CONTRIBUTIONS

*Study design, manuscript writing, data collection and statistical analysis*: Clément Favroul. *Study design and manuscript editing*: Robert A. Magnussen. *Study design and manuscript editing*: Cécile Batailler. *Manuscript editing*: Sébastien Lustig. *Study design, supervision, literature review and manuscript editing*: Elvire Servien. All authors read and approved the final manuscript.

## CONFLICT OF INTEREST STATEMENT

Cécile Batailler: Consultant for Stryker, Smith & Nephew. Sébastien Lustig: Consultant for Stryker, Smith & Nephew, Heraeus, Depuy Synthes; Institutional research support from Groupe Lepine, Amplitude; Editorial Board for *Journal of Bone and Joint Surgery (Am)*. Elvire Servien: Consultant for Corin. The remaining authors declare no conflicts of interest.

## ETHICS STATEMENT

All procedures were performed in accordance with the ethical standards of the institutional and/or national research committee, the 1964 Helsinki Declaration and its later amendments, or comparable ethical standards. Data collection and analysis were carried out in accordance with MR004 Reference Methodology from the Commission Nationale de l'Informatique et des Libertés (Ref. 2229975V0) obtained on 6 May 2023. The study was registered and filed on the Health Data Hub website. As per institutional standards, formal patient consent is not required for this type of study.

## Data Availability

Data are available on request due to privacy/ethical restrictions.
